# Evaluating a non-pharmacological treatment for primary premature ejaculation patients with low sexual frequency: a retrospective study on vacuum negative pressure hydropneumatic/pneumatic bubble massage

**DOI:** 10.1093/sexmed/qfaf069

**Published:** 2025-09-16

**Authors:** Qing-Qiang Gao, Chao-Ba He, Bin Wang, Yu-Tian Dai, Bai-Bing Yang, Xiao-Zhi Zhao

**Affiliations:** Department of Andrology, Nanjing Drum Tower Hospital, The Affiliated Hospital of Nanjing University Medical School, Nanjing, 210008, China; Department of Andrology, Guangdong Hospital of Traditional Chinese Medicine, Zhuhai, 519015, China; Department of Andrology, Nanjing Drum Tower Hospital, The Affiliated Hospital of Nanjing University Medical School, Nanjing, 210008, China; Department of Andrology, Nanjing Drum Tower Hospital, The Affiliated Hospital of Nanjing University Medical School, Nanjing, 210008, China; Department of Andrology, Nanjing Drum Tower Hospital, The Affiliated Hospital of Nanjing University Medical School, Nanjing, 210008, China; Department of Andrology, Nanjing Drum Tower Hospital, The Affiliated Hospital of Nanjing University Medical School, Nanjing, 210008, China

**Keywords:** vacuum negative pressure hydropneumatic/pneumatic bubble massage, premature ejaculation, low-frequency, intravaginal ejaculation latency time

## Abstract

**Background:**

Premature ejaculation (PE) is a common male sexual dysfunction with treatment limitations including side effects and partner dependency.

**Aim:**

To evaluate vacuum negative pressure hydropneumatic/pneumatic bubble massage (VNPHP/PBM) efficacy in primary PE (PPE) patients stratified by sexual frequency, focusing on subjective low-frequency (avoidance due to PE) vs objective low-frequency subgroups.

**Methods:**

Retrospective analysis of 42 PPE patients: 22 low-frequency (LF; <4 intercourse/month) including 13 subjective (sub-LF) and 9 objective (ob-LF) vs 20 non-low-frequency (NLF; ≥4/month).

**Outcomes:**

Primary: intravaginal ejaculation latency time (IELT); secondary: Premature Ejaculation Diagnostic Tool (PEDT), Self-Rating Anxiety Scale (SAS) scores, and sexual frequency changes at 4 weeks.

**Results:**

Both groups showed significant improvements in IELT, PEDT, and SAS scores (*P* < .05). Low-frequency group showed greater improvements than NLF in PEDT reduction (6.36 ± 2.38 vs 7.90 ± 2.02, *P* = .03), SAS reduction (30.95 ± 9.57 vs 38.45 ± 8.85, *P* = .01), and sexual frequency increase (0.50 [0.00, 4.00] vs 1.00 [1.00, 2.00], *P* = .02). Crucially, sub-LF patients exhibited dramatic sexual frequency normalization (6.00 [4.00, 7.50] vs 2.00 [1.00, 2.00], *P* < .001), while ob-LF unchanged (*P* = .56). No adverse events.

**Clinical implications:**

Vacuum negative pressure hydropneumatic/pneumatic bubble massage is a partner-independent therapy that not only improves ejaculatory control but also restores sexual activity in patients avoiding intercourse due to PE-related anxiety.

**Strengths and limitations:**

Strengths: First study analyzing subjective vs objective low-frequency PE, standardized protocols. Limitations: Retrospective design, self-reported IELT data, lack of a control group, and the non-blinded nature of the study.

**Conclusion:**

Vacuum negative pressure hydropneumatic/pneumatic bubble massage significantly improves PE symptoms with amplified benefits in low-frequency patients, particularly those with PE-driven sexual avoidance.

## Introduction

Premature ejaculation (PE) is one of the most common male sexual dysfunctions, characterized by a shortened intravaginal ejaculation latency time (IELT), a lack of control over ejaculation, and negative interpersonal results.^1^ According to the American Urological Association/Sexual Medicine Society of North America (AUA/SMSNA) and International Society for Sexual Medicine (ISSM) guidelines, the prevalence of both acquired and lifelong PE in the general population is ~5%, consistent with epidemiological data indicating that roughly 5% of men exhibit an IELT of less than 2 min.^2^ Premature ejaculation is typically categorized based on symptom onset: primary PE (PPE), where ejaculation occurs always or almost always before penetration or within about 1 min of vaginal insertion, and secondary PE, characterized by a shortened ejaculation latency of less than 3 min.^1^ The current standard treatments for PE include local anesthetics, selective serotonin reuptake inhibitors (SSRIs), including dapoxetine, and behavioral therapy (BT).^3^^,^^4^ However, these treatments are often associated with adverse reactions, high costs, suboptimal efficacy, and the need for pre-intercourse dosing, which results in poor patient compliance and high rates of treatment discontinuation.^3^

PE is traditionally attributed to both psychological and biological factors. Non-organic factors contributing to PE include psychological stress, lack of sexual techniques, early sexual experiences, and infrequent sexual intercourse.^1^Multiple studies suggest that BT could be an effective treatment for PE.^5-7^ The most commonly recommended behavioral strategies are the “Stop-Start” method and the “Squeeze” technique.^8^ The primary advantage of BT is that patients can learn techniques to delay ejaculation, overcome negative emotions, and experience less side effects.^9^ Research shows that BT can increase IELT of PE patients.^7^ It addresses aspects of PE that medications alone cannot solve and offers the potential for lasting results after discontinuing medication.^10^ However, BT requires both partners’ involvement, limiting its applicability for patients without stable partners or those who prefer not to involve their partners in treatment.^11^ Furthermore, both the “Stop-Start” and “Squeeze” techniques involve the patient or partner controlling ejaculation through masturbation, which may not fully replicate the sensations of sexual intercourse.^12^ The therapeutic benefits of BT may therefore require further trials to confirm its effectiveness.^11^

The combination of a masturbation aid device (eg, men’s training cup) and BT presents a new approach to treating PE.^11^^,^^13^ This method could offer longer IELT without side effects and stable results over time, making it a promising alternative to drug therapy.^11^ However, previous studies have not addressed potential differences in treatment outcomes based on individuals’ frequency of sexual intercourse, which may be closely related to PE.^11^^,^^14^ We hypothesize that the vacuum negative pressure hydropneumatic/pneumatic bubble massage (VNPHP/PBM), designed to simulate vaginal sensations, provides a structured and partner-independent training modality for patients with PE and low sexual frequency. To test this hypothesis, we aim to address the following research questions: (1) Does VNPHP/PBM-mediated training in a simulated vaginal environment significantly improve ejaculatory control and reduce anxiety symptoms in PE patients? (2) Do low-frequency PE patients exhibit significantly greater improvements in IELT and Premature Ejaculation Diagnostic Tool (PEDT) scores after VNPHP/PBM intervention compared to non-low-frequency PE patients? (3) Among low-frequency PE patients, do those with subjectively reduced intercourse frequency due to PE demonstrate significantly larger increases in sexual frequency post-treatment than those with objectively limited opportunities?

## Materials and methods

### Study design and patient selection

We retrospectively analyzed the clinical data of male patients with PPE who had received VNPHP/PBM treatment at Nanjing Drum Tower Hospital, Affiliated Hospital of Medical School, Nanjing University (Nanjing, China) between January 2020 and April 2024. According to the latest definition from the ISSM, PPE is characterized by the consistent occurrence of ejaculation before or within about 1 min of vaginal penetration during the first sexual experience.^1^ The AUA/SMSNA guidelines define PPE as “poor ejaculatory control, associated distress, and intravaginal ejaculation occurring within ~2 min following the first sexual experience”.^2^ Clinical and questionnaire data were collected, including baseline information, such as age, height, weight, marital status, history of PE, IELT, PEDT scores, International Index of Erectile Function (IIEF-EF) scores, Self-Rating Anxiety Scale (SAS) scores, and the monthly frequency of sexual intercourse. Data evaluation was performed by two experienced specialists. All patients were treated by the same sex therapist. The number of standard cases determines the sample size. This study was conducted in accordance with the Declaration of Helsinki and its subsequent amendments. Written informed consent was obtained from all participants for the use of their data in this research. The Institutional Review Board of Nanjing Drum Tower Hospital granted a waiver for formal ethical approval as the analysis utilized fully anonymized retrospective data. This study adhered to the STROBE guidelines for reporting observational studies.

Based on the widely adopted empirical criterion for “regular sexual activity” in international studies, defined as sexual intercourse ≥1 time per week (approximately ≥4 times per month), patients were stratified into two groups according to intercourse frequency: the low-frequency group (no more than 4 instances of sexual intercourse per month, Group LF) and the non-low-frequency group (no less than 4 instances of sexual intercourse per month, Group NLF).^15^^,^^16^ Group LF was further divided into two subgroups according to the reason of low sexual frequency. Subgroup sub-LF consisted of men who subjectively avoided sexual contact due to PE, despite having a partner, while subgroup ob-LF included those with objective reasons that leading to a low frequency of sexual intercourse (such as partners living in different locations or not yet being married).

Inclusion criteria: (1) Individuals suffering from PPE (Given the self-reported IELT data in this study, PPE patients were selected based on the diagnostic criteria of both AUA/SMSNA and ISSM guidelines, requiring IELT <2 min^1^^,^^2^); (2) heterosexuality; (3) normal sexual desire, defined as: (i) Presence of spontaneous sexual thoughts/fantasies (≥1 episode/week without external triggers), and (ii) absence of distress related to libido, as per the International Consultation on Sexual Medicine (ICSM) clinical principle for excluding hypoactive sexual desire dysfunction^17^; (4) normal erectile function (IIEF-EF ≥ 22); (5) PEDT score ≥ 11; (6) Stable heterosexual relationship with the same sexual partner for at least 6 months; (7) partner’s sexual function is normal and willing to cooperate in attempting sexual intercourse.

Exclusion criteria: (1) Other sexual dysfunctions apart from PE; (2) history of mental illness; (3) endocrine and cardiovascular diseases, or significant liver or kidney dysfunction that may affect ejaculation; (4) patients with partners who have poor relationships and refuse to engage in sexual activity or follow-up appointments; (5) patients with incomplete follow-up data.

To evaluate real-world treatment adherence, we analyzed all patients initiating VNPHP/PBM therapy between January 2020 and April 2024 (*N* = 262), regardless of diagnosis. Treatment completion required attendance at all 15 scheduled sessions. Premature discontinuation (<15 sessions) occurred in 42 patients (16.03%), primarily due to scheduling conflicts (*n* = 22), perceived lack of efficacy (*n* = 4), and loss to follow-up (*n* = 16). Notably, the discontinuation rate differed significantly between diagnostic groups: 19.70% (13/66) in the PE group (due to scheduling conflicts (*n* = 5), perceived lack of efficacy (*n* = 2), and loss to follow-up (*n* = 6)) vs 8.70% (10/115) in the primary intravaginal anejaculation (PIAJ) group (*P* < .001).

### VNPHP/PBM treatment

Vacuum negative pressure hydropneumatic/pneumatic bubble massage treatment was administered by certified sex therapists (accredited by the Chinese Sexology Association) using a male external genital therapy device (Model: SW-3503, Jiangsu Sanwei Medical Technology Co., Ltd.). The device is a Class III medically certified product (NMPA Approval No.: 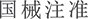 20143092258), with validated safety protocols, including biocompatibility, electrical safety (GB 9706.1), and operational performance, under simulated conditions prior to clinical deployment. During treatment, patients first gently pulled the bilateral testes downward to prevent accidental suction into the cylinder. After displacing pubic hair, the therapy cylinder was vertically positioned (90° angle) against the pubic symphysis to ensure sealing. The penis was then immersed in temperature-controlled water (37-39°C) simulating vaginal conditions to enhance efficacy and comfort. Initial negative pressure was set at 0.01-0.03 kPa based on individual tolerance, with patients stabilizing the cylinder at mid-section using the left hand to prevent slippage while maintaining visual monitoring of genitalia within the cylinder, and operating the pressure/bubble regulator with the right hand. Crucially, patients self-adjusted pressure/bubble intensity upon sensing the premonitory ejaculatory phase to emulate the “stop-start” technique. Following each 5-min device session, 3 min of pelvic floor muscle training was performed (Kegel exercises: 3 sets × 10 contractions with 5-s holds, involving rhythmic lifting and tightening of the pelvic floor muscles, including the anal sphincter). Therapists dynamically optimized parameters via concealed monitoring; unintended ejaculation triggered immediate cessation of stimulation, followed by drainage of seminal fluid-contaminated water and replacement with fresh saline. After a 30-min rest, treatment resumed with calibrated parameters: reduced negative pressure (0.015-0.02 kPa), decreased bubble intensity (Level 1-2), and adjusted water temperature (35-36°C). The full protocol comprised 15 twice-weekly sessions (20 min/session), with real-time parameter optimization guided by patient tolerance feedback. Contraindications included: (1) genital trauma or congenital malformations, (2) active genital inflammation, (3) phimosis or preputial stenosis, (4) coagulation disorders, and (5) severe systemic diseases, substance/alcohol abuse within 6 months, long-term antipsychotic medication use, or psychiatric disorders.

### Efficacy evaluation and observation indicators

The treatment outcomes were evaluated based on patient records 4 weeks after treatment completion. The primary measure was the change in IELT, while secondary measures included PEDT score, SAS score, and changes in sexual intercourse frequency. Documented adverse events during the treatment period were also analyzed.

### Follow-up

Follow-up data were obtained from patient records or documented telephone consultations conducted during the 4th week after treatment. If patients did not attend scheduled follow-up appointments in person, their data from telephone follow-ups were retrospectively reviewed.

### Statistical analysis

Data were analyzed using the Statistical Program for Social Sciences (SPSS) software (version 25.0; IBM Corp.). Categorical variables were expressed as frequency and rate, with differences analyzed using the χ^2^ test or Fisher’s exact test. Normality and equality of variance were assessed using the Shapiro–Wilk test and Levene’s test, respectively. If normality and homogeneity of variance were confirmed, group differences were analyzed using the independent samples *t*-test, and intragroup differences were examined using paired *t*-tests. Continuous variables were expressed as mean ± SD. Otherwise, non-normally distributed continuous variables are reported as median (interquartile range) and analyzed using Mann–Whitney U tests (between groups) or Wilcoxon signed-rank tests (within groups). A significance level of *P* < .05 was used for all analyses.

## Results

### Baseline conditions of the two groups of patients

**Figure 1:**
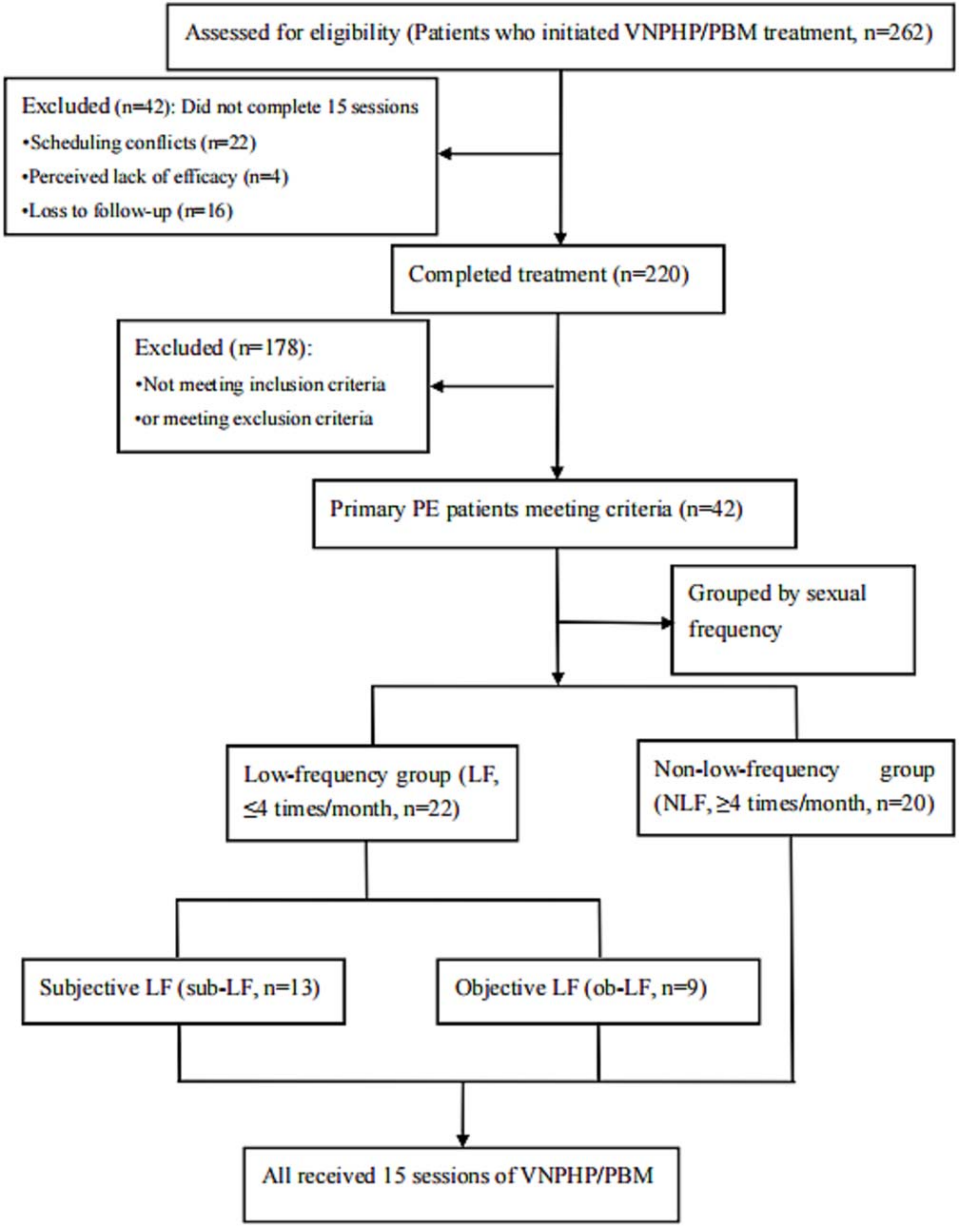
Flowchart of patient enrollment. VNPHP/PBM, vacuum negative pressure hydropneumatic/pneumatic bubble massage; PE, premature ejaculation.

Based on the inclusion and exclusion criteria, the data of 42 patients were collected. The flow diagram is shown in Figure 1. Among them, 22 patients were categorized as low-frequency sexual intercourse group (Group LF), of whom 13 had subjective reasons (Group sub-LF) and 9 had objective reasons (Group ob-LF). The other 20 patients were categorized as non-low-frequency group sexual intercourse group (Group NLF). All patients received 15 cycles of VNPHP/PBM treatment, with the last follow-up date being May 10, 2024. The baseline characteristics of the patients are shown in Table 1. There were no significant differences between the two groups in age, height, weight, marital status, history of PE, PEDT score, SAS score, IIEF-EF scores, and IELT (all *P* > .05). The frequency of sexual intercourse in Group LF was significantly lower than that in Group NLF (*P* < .001) (Table 1).

**Table 1:** Baseline conditions of the two groups of patients.

	Group LF (*n* = 22)	Group NLF (*n* = 20)	*P*-value
Age (years)	29.50 (26.75, 36.75)	34.50 (28.50, 43.25)	.12
Height (cm)	176.73 ± 3.34	175.85 ± 5.88	.56
Weight (kg)	81.75 (69.75, 90.50)	74.00 (70.00, 92.50)	.52
Marital status, *n* (%)			.54
Married	10 (45.45)	11 (55.00)	
Unmarried	12 (54.55)	9 (45.00)	
PE duration (month)	24.00 (11.00, 39.00)	21.00 (12.00, 28.50)	.58
IIEF-EF score	27.00 (27.00, 29.00)	27.00 (26.00, 28.00)	.24
PEDT score	14.50 (13.00, 16.00)	15.00 (12.25, 17.00)	.55
SAS score	47.41 ± 8.42	45.45 ± 8.27	.45
Self-estimated IELT, *n* (%)			.81
0-0.5	9 (40.91)	8 (40.00)	
0.6-1	8 (36.36)	8 (40.00)	
1.1-2	5 (22.72)	4 (20.00)	
Sexual intercourse frequency (/month)	1.00 (1.00, 2.00)	6.00 (4.00, 9.50)	<.001

Group LF: low frequency of sexual intercourse group; Group NLF: non-low-frequency of sexual intercourse group.

Values are presented as mean ± SD, median (interquartile range), or number (percent).

Abbreviations: IELT, intravaginal ejaculation latency time; IIEF-EF, International Index of Erectile Function; PE, premature ejaculation; PEDT, Premature Ejaculation Diagnostic Tool; SAS, Self-Rating Anxiety Scale.

### Comparisons of therapeutic effects between groups

Both groups showed significant improvements in IELT, PEDT score and SAS score at 4 weeks post-treatment (all *P* < .05). Additionally, the frequency of sexual intercourse significantly increased (Group LF, *P* < .01; Group NLF, *P* = .03) (Table 2). After VNPHP/PBM treatment, Group LF showed a higher PEDT score (6.36 ± 2.38 vs 7.90 ± 2.02, P = 0.03), a lower SAS score (30.95 ± 9.57 vs 38.45 ± 8.85, *P* = .01); and a more significant increase in the frequency of sexual intercourse (0.50 (0.00, 4.00) vs 1.00 (1.00, 2.00), *P* = .02) when compared with Group HF (Table 2).

**Table 2:** Comparisons of therapeutic effects between groups.

	Baseline		4 weeks post-treatment	
Group	LF (*n* = 22)	NLF (*n* = 20)	LF (*n* = 22)	NLF (*n* = 20)
IELT (min)	1.00 (0.50, 1.63)	1.00 (0.50, 1.38)	4.00 (4.00, 5.00)^a^	4.00 (4.00, 4.00)^a^
PEDT score	14.64 ± 1.87	15.05 ± 2.33	6.36 ± 2.38^a^	7.90 ± 2.02^ab^
SAS score	47.41 ± 8.42	45.45 ± 8.27	30.95 ± 9.57^a^	38.45 ± 8.85^ab^
Sexual intercourse frequency (/month)	1.00 (1.00, 2.00)	6.00 (4.00, 9.50)^b^	3.50 (1.00, 6.25)^a^	8.00 (6.00, 9.50)^ab^
Sexual intercourse frequency change			0.50 (0.00, 4.00)	1.00 (1.00, 2.00)^b^

Group LF: low frequency of sexual intercourse group; Group NLF: non-low-frequency of sexual intercourse group.

Compared with baseline, ^a^*P* < .05.

Compared with Group LF, ^b^*P* < .05.

Values are presented as mean ± SD or median (interquartile range).

Abbreviations: IELT, intravaginal ejaculation latency time; PEDT, Premature Ejaculation Diagnostic Tool; SAS, Self-Rating Anxiety Scale.

### Comparison of outcomes in patients from sub-LF and ob-LF subgroups before and after VNPHP/PBM treatment

Before treatment, there were no significant differences between the two subgroups in terms of IELT, PEDT score, SAS score and sexual intercourse frequency (*P* > .05). After treatment, except for sexual intercourse frequency, all the above indicators in both subgroups were significantly improved (*P* < .05), but there were no significant differences between the two subgroups (*P* > .05). The sexual intercourse frequency in Group sub-LF was significantly increased compared with that before treatment (6.00 (4.00, 7.50) vs 2.00 (1.00, 2.00), *P* < .001), and was significantly higher than that in Group ob-LF (6.00 (4.00, 7.50) vs 1.00 (1.00, 2.00), *P* < .001). In contrast, the sexual intercourse frequency in Group ob-LF did not show significant changes (1.00 (1.00, 1.50) vs 1.00 (1.00, 2.00), *P* = .56) (Table 3).

**Table 3:** Comparison of outcomes in patients from sub-LF and ob-LF subgroups before and after VNPHP/PBM treatment.

	Before treatment		4 weeks post-training	
Subgroup	sub-LF (*n* = 13)	ob-LF (*n* = 9)	sub-LF (*n* = 13)	ob-LF (*n* = 9)
IELT (min)	1.00 (0.50, 1.75)	1.00 (0.50, 1.50)	4.00 (3.00, 5.00)^a^	4.00 (4.00, 5.00)^a^
PEDT score	15.15 ± 2.15	13.89 ± 1.05	6.31 ± 2.10^a^	6.44 ± 2.88^a^
SAS score	49.08 ± 8.19	45.00 ± 8.62	31.77 ± 9.88^a^	29.78 ± 9.58^a^
Sexual intercourse frequency (/month)	2.00 (1.00, 2.00)	1.00 (1.00, 1.50)	6.00 (4.00, 7.50)^a^	1.00 (1.00, 2.00)^b^

Group sub-LF: subjective low-frequency sexual intercourse PE group; Group ob-LF: objective low-frequency sexual intercourse PE group.

Compared with before treatment, ^a^*P* < .05.

Compared with Group sub-LF, ^b^*P* < .05.

Values are presented as mean ± SD or median (interquartile range).

Abbreviations: IELT, intravaginal ejaculation latency time; PEDT, Premature Ejaculation Diagnostic Tool; SAS, Self-Rating Anxiety Scale.

## Discussion

This study demonstrates that VNPHP/PBM significantly improves ejaculatory control by training patients within a simulated vaginal environment. This training enhances self-confidence and facilitates the transfer of acquired skills to actual sexual encounters. Men with low-frequency PE derived greater benefit from this intervention, irrespective of whether the low frequency stemmed from subjective or objective causes. Importantly, patients exhibiting subjectively reduced intercourse frequency due to PE-related avoidance (sub-LF subgroup) demonstrated the most pronounced improvement in coital frequency (6.00 [4.00, 7.50] vs 2.00 [1.00, 2.00], *P* < .001) alongside significantly reduced anxiety levels (*P* < .001) (Table 3). This positions VNPHP/PBM as a novel, non-pharmacological treatment option particularly well-suited for this specific low-frequency PE patient subgroup.

Ejaculation involves coordination between multiple systems, including the central nervous system and the ejaculation generator in the spinal cord.^18^ Human sexual activity is influenced by biological, psychological, and social factors.^19^ Although the exact cause of PE is unclear, it is generally considered a sexual psychological disorder with both psychogenic and organic causes.^1^ Standard treatments include BT, local anesthetics, and oral pharmacotherapy.^3^ Selective serotonin reuptake inhibitors, commonly used to treat PE, work by increasing serotonin levels to prolong IELT but have notable side effects like nausea, headaches, and dizziness, leading many patients to discontinue treatment.^20^ Local anesthetic treatments for PE, though effective, can cause side effects like erectile dysfunction and decreased penile sensation.^4^^,^^9^ In contrast, VNPHP/PBM offers an alternative with no adverse effects and the potential for sustained benefits.

Previous studies have indicated that masturbation aid devices can improve IELT in patients with PE, yet they are limited by issues, such as high dropout rates, due to inadequate environmental simulation and lack of professional guidance.^11^^,^^13^ In contrast to the traditional stop-start method, which lacks environmental standardization, and commercially available masturbation aids that typically provide only static stimulation tiers without thermal modulation or real-time biofeedback capabilities, VNPHP/PBM delivers distinct therapeutic advantages under clinician supervision. VNPHP/PBM utilizes hydropneumatic/pneumatic bubbles within a temperature-controlled water environment (37-39 °C) combined with adjustable negative pressure (0.01-0.03 kPa), enabling dynamic, patient-directed, and graded real-time modulation of tactile and thermal stimuli to simulate physiological intravaginal dynamics. Patients can actively and in real-time self-regulate bubble intensity during the premonitory sensation (sense of urgency) preceding ejaculation, practicing the stop-start technique and thereby enhancing contextual relearning. This active modulation facilitates the understanding of one’s own ejaculatory inhibition threshold and boosts self-efficacy, standing in sharp contrast to the traditional stop-start method characterized by non-standardized stimulation and abrupt cessation. Importantly, the subjective LF subgroup exhibited both significantly increased sexual frequency (*P* < .001) and reduced SAS scores (*P* < .001), indicating diminished PE-related avoidance behavior and performance anxiety. This dual improvement strongly suggests enhanced capacity for partnered sexual engagement. Furthermore, the VNPHP/PBM protocol incorporates structured pelvic floor muscle training (Kegel exercises during rest intervals) and therapist-guided parameter optimization (eg, post-ejaculatory pressure/temperature adjustments). Patients receive graded penile stimulation under therapist guidance, allowing for real-time parameter adjustments to optimize treatment efficacy. This therapist-supported approach demonstrated broader clinical utility in reducing patient attrition. Analysis of all PE patients receiving VNPHP/PBM therapy at our center showed a dropout rate of 19.70% (13/66), while the dropout rate for self-operated masturbation aid devices was 51.35% (19/37).^13^ This synergy of standardized stimulation and real-time professional guidance is paramount for ensuring the consistent implementation of the treatment protocol. Crucially, the clinical deployment of VNPHP/PBM ensures safety monitoring, standardized stimulus delivery, and the synergistic integration of multimodal therapy components. This clinical necessity justifies its use beyond unassisted application of commercial devices or self-administered behavioral therapy, offering enhanced safety and therapeutic outcomes.

Interestingly, previous research has demonstrated the therapeutic efficacy of masturbation aid devices in patients with intravaginal ejaculatory dysfunction (also known as PIAJ).^21^^,^^22^ The therapeutic efficacy of VNPHP/PBM across opposing ejaculatory dysfunctions stems not from altering penile sensitivity but from its core function as a behavioral simulator that addresses shared psychogenic factors.^15^ For PE, the device provides graded vaginal-environment simulation combined with therapist-guided “stop-start” therapy setting. For PIAJ, the same simulation reduces performance pressure while recalibrating central processing of sexual stimuli through exposure to intercourse-like sensations, particularly targeting stimulus-requirement mismatch resulting from special self-masturbation patterns (maladaptive overlearned behaviors).^15^ Crucially, both applications leverage contextual relearning: by decoupling ejaculation from prior maladaptive associations (eg, anxiety-driven urgency in PE or overlearned masturbatory patterns in PIAJ, VNPHP/PBM facilitates transfer of skills to real intercourse. Notably, lower discontinuation rates were observed in PIAJ vs PE patients (8.70% vs 19.70%; *P* < .001). This divergence likely reflects stronger treatment motivation in PIAJ cohorts due to limited therapeutic alternatives, contrasting with PE patients’ access to multiple intervention options.

To our knowledge, this is the inaugural study to assess the efficacy and safety of VNPHP/PBM in low-frequency PE patients (subjective or objective reasons). However, this study has several limitations. First, as a retrospective investigation, the absence of a control group and blinding may introduce selection bias and measurement bias, necessitating validation through future multicenter prospective randomized controlled trials (RCTs). Second, the relatively small sample size may constrain the generalizability of the findings. Third, partial data collection via telephone interviews carries risks of recall bias and social desirability bias. Fourth, self-reported IELT was used instead of stopwatch measurement to avoid disrupting the natural sexual experience; however, this approach may introduce reporting bias. Nevertheless, self-reported IELT is an established method in clinical practice and aligns with the retrospective design of this study, though objective measurement would improve scientific rigor in future research.^23^ Fifth, sexual frequency metrics may be subject to ceiling effects in high-activity cohorts, potentially underestimating therapeutic benefits in these patients when using change-from-baseline analyses. Future trials should incorporate metrics less susceptible to ceiling effects (eg, sexual satisfaction scales) to complement frequency measures. Finally, the 4-week follow-up period precludes assessment of long-term efficacy.

## Conclusion

This study demonstrates that VNPHP/PBM is an effective non-pharmacological intervention for PPE, particularly benefiting patients with low sexual frequency. The therapy significantly improves ejaculatory control, alleviates PE-related anxiety and distress symptoms, and restores sexual activity—particularly for patients avoiding intercourse due to PE-related psychological factors. The therapy’s partner-independent nature and absence of adverse events position it as a clinically valuable option for PE management. Future multicenter prospective studies with larger cohorts, objective IELT measurement, and long-term follow-up are warranted to validate these findings and optimize treatment protocols.

## Data Availability

The datasets used and/or analyzed during the current study available from the corresponding author on reasonable request.
